# H3K9me3 facilitates hypoxia-induced p53-dependent apoptosis through repression of APAK

**DOI:** 10.1038/onc.2015.134

**Published:** 2015-05-11

**Authors:** M M Olcina, K B Leszczynska, J M Senra, N F Isa, H Harada, E M Hammond

**Affiliations:** 1 Department of Oncology, CR-UK & MRC Oxford Institute for Radiation Oncology, University of Oxford, Oxford, UK; 2 Department of Radiation Oncology and Image-Applied Therapy, Kyoto University Graduate School of Medicine, Kyoto, Japan

## Abstract

Regions of hypoxia occur in most solid tumors, and they are associated with a poor prognostic outcome. Despite the absence of detectable DNA damage, severe hypoxia (<0.1% O_2_) induces a DNA damage response, including the activation of p53 and subsequent induction of p53-dependent apoptosis. Factors affecting hypoxia-induced p53-dependent apoptosis are unclear. Here we asked whether H3K9me3, through mediating gene repression, could regulate hypoxia-induced p53-dependent apoptosis. Under hypoxic conditions, increases in H3K9me3 occur in an oxygen-dependent but HIF-1-independent manner. We demonstrate that under hypoxic conditions, which induce p53 activity, the negative regulator of p53, APAK, is repressed by increases in H3K9me3 along the *APAK* loci. *APAK* repression in hypoxia is mediated by the methyltransferase SETDB1 but not Suv39h1 or G9a. Interestingly, increasing hypoxia-induced H3K9me3 through pharmacological inhibition of JMJD2 family members leads to an increase in apoptosis and decreased clonogenic survival and again correlates with APAK expression. The relevance of understanding the mechanisms of APAK expression regulation to human disease was suggested by analysis of patients with colorectal cancer, which demonstrates that high APAK expression correlates with poor prognosis. Together, these data demonstrate the functional importance of H3K9me3 in hypoxia, and they provide a novel mechanistic link between H3K9me3, p53 and apoptosis in physiologically relevant conditions of hypoxia.

## Introduction

Tumor hypoxia arises in the majority of solid tumors as a consequence of high metabolic demand for oxygen and rapid growth coupled to the inefficiency of new blood vessels formed by angiogenesis.^1^ The hypoxia-inducible factors (HIF) are typically regarded as the main drivers of the hypoxic response.^2^ Although HIF can be induced in relatively mild levels of hypoxia, severe levels of hypoxia (<0.1% O_2_) are required to induce a DNA damage response, the downstream effect of which is p53 activation.^3^ In response to severe hypoxia, replication stress occurs, leading to increased activity of both ATR and ATM, which in turn phosphorylate p53 at serine 15 leading to increased p53 stability/activity and the subsequent induction of p53-dependent apoptosis.^4, 5, 6^ Activation of the DNA damage response has been proposed to contribute to an early barrier to tumorigenesis by inducing p53-mediated senescence or apoptosis.^7, 8^ Hypoxia therefore drives the selection pressure for mutation of p53 or inactivation of the p53 pathway.^9^

In unstressed conditions, p53 protein is kept at low levels through proteasomal degradation, but it can be rapidly stabilized and activated in response to a wide variety of stresses. In addition, the activity and function of low constitutive levels of p53 are subject to further levels of control. *APAK* (ATM and p53-Associated KZNF Protein/ZNF420) encodes a KZNF protein that binds to p53 through its KRAB domain in unstressed cells.^10^ APAK binding attenuates acetylation of p53 by recruiting KAP-1-HDAC1 complexes, and it negatively regulates p53. Upon DNA damage, ATM-dependent phosphorylation of p53, KAP-1 and APAK results in dissociation of the APAK-p53 complex, favoring the transcriptional activation of proapoptotic genes by p53.^11^ In addition, APAK can prevent p53 from binding to the proapoptotic gene *p53AIP1* by binding to a consensus sequence (TCTTN2−30TTGT) overlapping the p53-binding site.^12^

The regulation of chromatin structure is essential for a number of nuclear processes including transcription, replication and repair. Histones, for example, can be heavily post-translationally modified, leading to changes toward a generally repressive or activating state depending on the modification and the residue targeted.^13^ H3K9me3 is typically associated with heterochromatin and gene repression, whereas H3K9me1 and H3K9me2 are mostly found in euchromatic regions.^14, 15^ Regulation of H3K9 methylation is catalyzed by a number of histone methyltransferases and removed by demethylases. The main H3K9 methyltransferases include Suv39h1, Suv39h2, G9a, GLP/EuHMTase1 and SETDB1/ESET.^16, 17, 18^ The development of specific inhibitors to each of the H3K9me3 methyltransferases has proven to be challenging to date; however, levels of this mark can be modulated through pharmacological inhibition of the relevant demethylases.^19^ ML324 is a recently described inhibitor, which leads to increased histone methylation, including H3K9me3, through inhibition of the JMJD2 enzymes. ML324 exhibits inhibitory activity against JMJD2E in the submicromolar range, and it has demonstrated efficacy at reducing human cytomegalovirus, as well as herpes simplex infection.^19^

Increases in H3K9me3 and decreases in H3K9 acetylation have been reported in a number of cell lines exposed to hypoxia with the subsequent repression of a number of different genes, including some involved in DNA repair such as *RAD51, MLH1* and *BRCA1*.^20, 21, 22^ The majority of studies investigating hypoxia-induced chromatin changes have been conducted at levels of mild or moderate hypoxia (1–2% O_2_), where cells are able to proliferate normally. Little is known about the chromatin changes that occur in the severely hypoxic (<0.1% O_2_) regions of tumors associated with the most therapy-resistant/aggressive cell populations and p53 activation (radiobiologic hypoxia).^23^ Here, we investigated the impact of H3K9me3 on hypoxia-induced p53-dependent apoptosis under severely hypoxic conditions. We identified *APAK* as an H3K9me3-repressed gene in hypoxic conditions that also lead to p53 stabilization. We demonstrated that under hypoxic conditions H3K9me3 enrichment increases along the *APAK* loci leading to gene repression in a SETDB1-dependent manner. The biological consequence of decreased levels of APAK is an increase in p53-dependent hypoxia-induced apoptosis. Most importantly, we show that increased H3K9me3 levels, decreased APAK expression and apoptosis can result from pharmacological inhibition of the JMJD2 demethylase. The relevance of understanding the mechanisms of APAK expression regulation to human disease was suggested by analysis of a small set of patients with colorectal cancer, which demonstrates that high APAK expression correlates with poor prognosis

## Results and Discussion

### APAK and p53-mediated apoptosis

**Figure 1:**
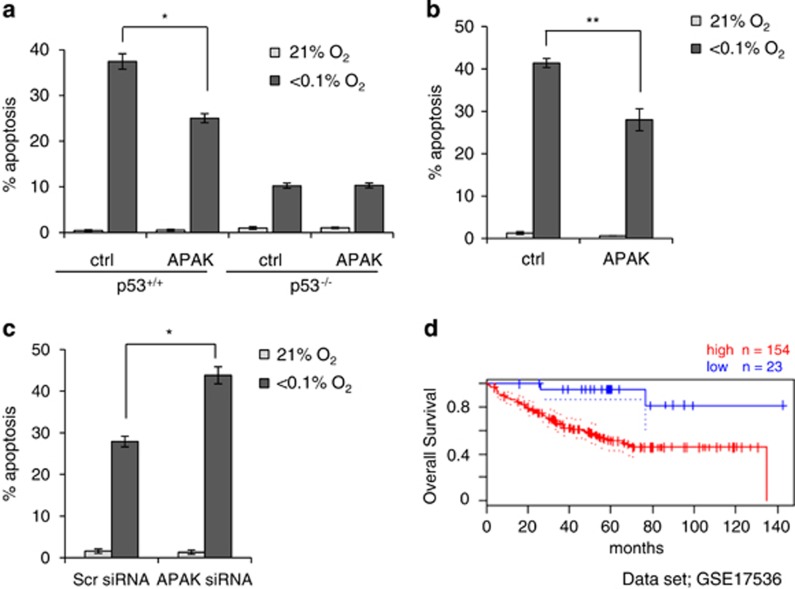
APAK and p53-mediated apoptosis. (**a**) Myc-tagged-APAK (Chunyan Tian, Beijing, China) (APAK) or Myc-empty plasmids (OriGene, Rockville, MD, USA) (Ctrl) were transfected into HCT116 p53^+/+^ or p53^−/−^ cells. These cells were then exposed to either Norm (21% O_2_) or Hyp (<0.1% O_2_ - 24 h). The graph represents the number of apoptotic/nonapoptotic cells expressed as a percentage of the whole population. Error bars indicate standard error between the 10 fields of view counted. Images were taken using a LSM780 (Carl Zeiss Microscopy Ltd, Jena, Germany) confocal microscope. A representative graph of one of three independent experiments is shown in each figure showing the percentage of apoptosis unless otherwise stated in the legend. (**b**) RKO cells transfected with either Myc-APAK or Myc-empty plasmids were exposed to Norm or Hyp for 18 h. Apoptosis was measured as in (**a**). (**c**) RKO cells were treated with Scramble or APAK siRNA (ON-TARGETplus SMARTpool, # L-016766-02-0005, Thermo Scientific) and exposed to Norm or Hyp for 18 h. Apoptosis was measured as in **(a**). (**d**) PrognoScan database-based Kaplan–Meier analysis of the overall survival of 177 colorectal cancer patients after the removal of primary tumors stratified by high (red: *n*=154) and low (blue: *n*=23) APAK levels. This analysis was based on the PrognoScan database (http://www.prognoscan.org/) using the publicly available Gene Expression Omnibus (http://www.ncbi.nlm.nih.gov/geo) with the accession numbers GSE 17536.^28, 29^

We hypothesized that APAK overexpression would lead to a decrease in apoptosis under hypoxic conditions and that this would occur in a p53-dependent manner. To test this hypothesis, we overexpressed APAK in both HCT116 p53^+/+^ and p53^-/-^ isogenic cell lines and exposed them to <0.1% O_2_. As expected, HCT116 p53^+/+^ underwent apoptosis in response to hypoxia, whereas this was diminished in the p53^-/-^ cells. As predicted, APAK overexpression significantly decreased the levels of hypoxia-induced p53-dependent apoptosis (Figure 1a and Supplementary Figure S1a). We also found that APAK overexpression led to a reduction in the number of apoptotic cells in the RKO (p53^+/+^) cell line (Figure 1b and Supplementary Figure S1b). We then carried out the reciprocal experiment and used siRNA to reduce APAK levels in both normoxia and hypoxia (<0.1% O_2_). Decreased expression of APAK was verified, and it led to a significant increase in apoptosis (Figure 1c and Supplementary Figure S1c). Together, these data demonstrate that APAK expression correlates with hypoxia-induced p53-dependent apoptosis. Notably, the relevance of APAK expression to human disease was suggested by analysis of a small set of patients with colorectal cancer, which demonstrates that high APAK expression correlates with poor overall survival (Figure 1d).

### APAK is repressed in hypoxia

**Figure 2:**
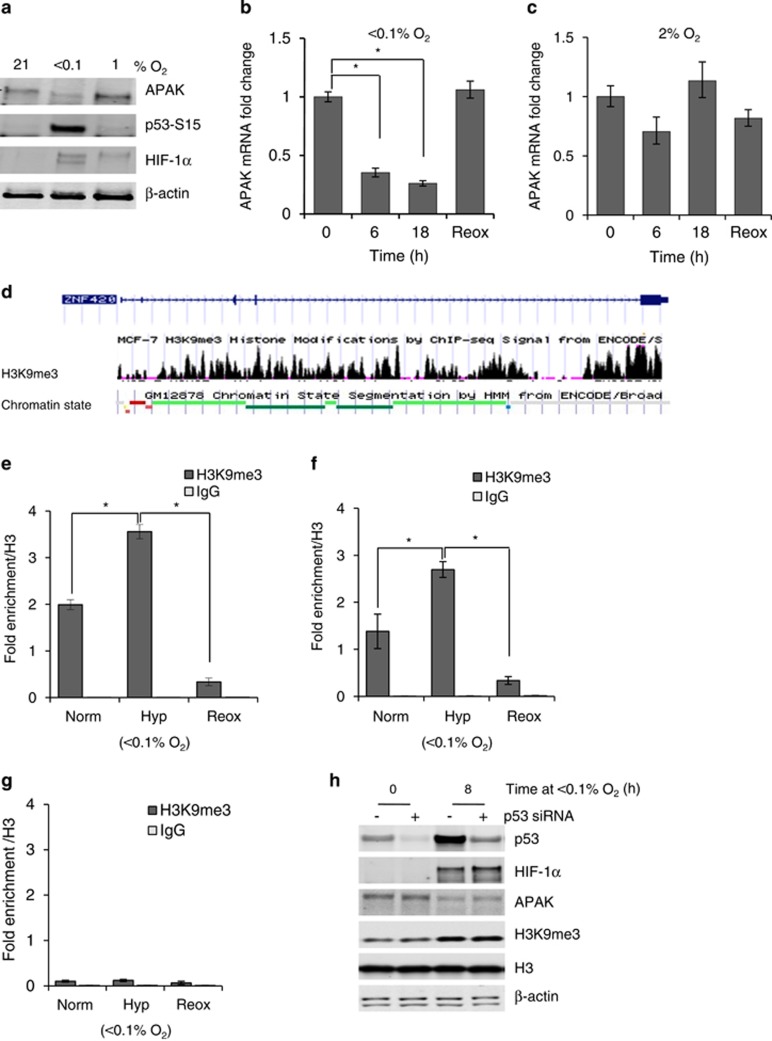
APAK is repressed in an oxygen-dependent manner. (**a**) RKO cells were exposed to 21, <0.1 or 1% O_2_ for 24 h, and western blotting was carried out with the indicated antibodies. Samples were collected in UTB buffer (9 M urea, 75 mM Tris-HCl pH 7.5 and 0.15 M β-mercaptoethanol). APAK (Sigma-Aldrich UK Prestige antibodies, Sigma-Aldrich, St Louis, MO, USA), p53-S15 (Cell Signaling, Danvers, MA, USA) and HIF-1α (Becton Dickinson Biosciences, San Jose, CA, USA) were used as markers of hypoxia, and β-actin (Santa Cruz Biotechnology, Dallas, TX, USA) was used as a loading control. The Odyssey infrared imaging technology (LI-COR Biotechnology Lincoln, NE, USA) was used (LI-COR Biosciences). All western blots shown are representative of one of three independent experiments unless otherwise stated. (**b**) The mRNA level of APAK was measured by qPCR (APAK forward: 5′-CAAAGGCAAGATGGAGAAGC-3′ and APAK reverse: 5′-TAGGTGTGAGGCTCGTCTGA-3′) in RKO cells exposed to <0.1% O_2_ for 0, 6, 18 h or Reox (6 h at <0.1% O_2_ followed by 2 h at 21% O_2_). Cells were then harvested in TRIzol (Invitrogen/Life Technologies, Grand Island, NY, USA) and RNA was extracted. cDNA was isolated and used for qPCR reactions using the SuperScript VILO kit (Invitrogen/Life Technologies) according to the manufacturer's recommendations. qPCR reactions were carried out using the 7500 Fast Real Time PCR System (Applied Biosystems, Grand Island, NY, USA). All mRNA levels shown were normalized to *18S* ribosomal RNA and calculated using a 2^−ΔΔCt^ method. Error bars indicate the error between the three technical replicates for each experiment ±RQ_max_ and RQ_min_ from one experiment. All experiments showing mRNA expression were carried out in triplicate unless otherwise stated. (**c**) mRNA level of APAK was measured by qPCR in RKO cells exposed to 2% O_2_ for 0, 6, 18 h or Reox (6 h at 2% O_2_ followed by 2 h at 21% O_2_), as described above. (**d**) Track of H3K9me3 binding along *APAK* from UCSC genome browser including a representative schematic of a possible set of chromatin state segmentation patterns (GRCh37/hg19 assembly). Chromatin state key: light gray (heterochromatin/low signal, repetitive/copy number variation), yellow (weak/poised enhancer), orange (strong enhancer), bright red (active promoter), light red (weak promoter), light green (weak transcribed), dark green (transcriptional transition/elongation) and blue (insulator). (**e**) and (**f**) RKO cells were exposed to Norm (21% O_2_), Hyp (6 h, <0.1% O_2_) or Reox (6 h, <0.1% O_2_ followed by 1 h of 21% O_2_). H3K9me3 fold enrichment/H3 at the *APAK* loci was assessed by ChIP followed by qPCR for each sample using primers designed to target a region in which H3K9me3 binding was expected (assembly used: GRCh37/hg19). Error bars indicate the standard error between technical replicates for one of three independent experiments. Primers used in (**e**): APAK ChIP forward: 5′-GTGTGGCAAGGCCTTTAGTC-3′, APAK ChIP reverse: 5′-GGGCTTCTCACCAGTATGGA-3′). Primers used in (**f**): (APAK ChIP2 Forward: 5′-TGGGAAAGCCTTTATTCGTG-3′, APAK ChIP2 Reverse: 5′-CTCCAGTGTGAATTCGCTGA-3′). (**g**) RKO cells were treated as in (b/c). The fold enrichment of H3K9me3 on a region along the *APAK* gene where minimal binding was expected was assessed by ChIP-qPCR, as previously described, with the following primers: (forward: 5′-GCTACAGCCTGCCTGGTATT-3′, reverse: 5′-ACACAAAACCACAGCCACAC-3′). (**h**) RKO cells were treated with Scramble (Stealth RNAi negative control (Invitrogen, Grand Island, NY, USA)) or p53 siRNA (5′-GUAAUCUACUGGGACGGAA-3′dTdT) (Ambion /Life Technologies, Grand Island, NY, USA) and exposed to 0 or 8 h of <0.1% O_2_. Western blotting was carried out with the antibodies indicated: p53 (Santa Cruz Biotechnologies) and H3 (Cell Signaling).

Given the observed role for APAK in inhibiting p53-dependent apoptosis, we speculated that APAK expression in hypoxia should be tightly regulated.^11^ Therefore, we assessed APAK protein levels under different oxygen tensions. We noted a marked reduction in protein expression after exposure to severe hypoxia (<0.1% O_2_), whereas no reduction in protein expression was observed following exposure to milder hypoxic conditions. The oxygen dependency of APAK repression correlated with the levels of oxygen that also lead to p53 activity (Figure 2a). Next, we examined the oxygen dependency of APAK mRNA expression by exposing RKO cells to <0.1 or 2% O_2_ for the times indicated. APAK mRNA expression was repressed at <0.1% O_2_ but not in the milder hypoxic conditions (Figures 2b and c).

We tested whether HIF-1 could be regulating APAK expression, and found that neither HIF-1α nor HIF-1β appeared to have a role in regulating APAK expression under <0.1% O_2_ (Supplementary Figures S2a–d). Epigenetic regulation is an other important mechanism of gene expression regulation in hypoxia.^6^ Given that we had found that APAK was repressed in an HIF-1-independent manner in hypoxia, we decided to check whether chromatin repressive marks such as H3K9me3 were reported as enriched along the *APAK* loci. Using the UCSC genome browser (GRCh37/hg19 genome assembly), we found an enrichment of the H3K9me3 chromatin mark along the *APAK* gene. Interestingly, we also found a large proportion of the *APAK* gene correlating with regions associated with heterochromatin as assessed by computationally integrating ChIP-seq data using a Hidden Markov Model^24^ (Figure 2d). This suggested that APAK is in a region of relatively inaccessible chromatin, and we subsequently verified this by EpiQ analysis (Supplementary Figure S2e). Importantly, this analysis demonstrated that the degree of chromatin compaction increased in response to hypoxia. Furthermore, ChIP-qPCR analysis of the *APAK* loci showed marked enrichment of H3K9me3 specifically in hypoxia (<0.1% O_2_), using two different sets of primers. Notably, this enrichment was decreased following reoxygenation mirroring the global changes in this histone mark previously assessed by western blotting (Figures 2e and f).^6^ ChIP analysis of a control region displaying minimal H3K9me3 on the UCSC browser profile, as expected, showed minimal H3K9me3 enrichment in normoxia, hypoxia and reoxygenation (Figure 2g). The observed specific transcriptional repression of APAK in response to <0.1% O_2_ was consistent with the oxygen-dependent regulation of H3K9me3, supporting the hypothesis that this histone modification is involved in repressing APAK. As p53 has recently been linked to H3K9me3 regulation, we investigated a potential role for p53 in the hypoxia induction of H3K9me3.^25^ siRNA-mediated knockdown of p53 had no effect on the hypoxic induction of H3K9me3 (Figure 2h). In support of the importance of H3K9me3 in APAK regulation in hypoxia, repression of APAK remained unchanged upon p53 loss.

### SETDB1 is involved in mediating APAK repression in hypoxia

**Figure 3:**
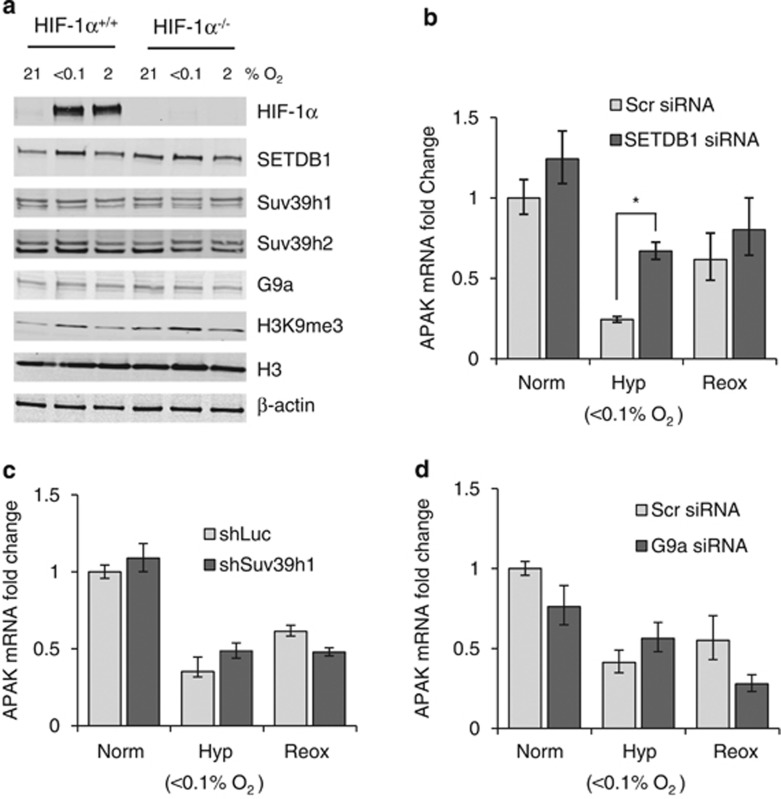
SETDB1 mediates APAK repression in hypoxia. (**a**) RKO HIF-1α^+/+^ and HIF1α^−/−^cells were exposed to 21%, 2% or <0.1% O_2_ for 6 h and western blotting was carried out with the antibodies indicated: Suv39h1 and Suv39h2 (Abcam, Cambridge, UK); SETDB1, G9a and H3 (Cell Signaling); and H3K9me3 (Upstate/Millipore, Billerica, MA, USA). (**b**) RKO cells were treated with Scramble (Stealth RNAi negative control (Invitrogen)) or SETDB1 siRNA (ON-TARGETplus SMARTpool, # L-020070-00-0005, Thermo Scientific) and exposed to 6 h of Norm (21% O_2_), Hyp (<0.1% O_2_) or Reox (6 h at <0.1% O_2_ followed by 2 h at 21% O_2_). APAK mRNA levels were assessed by qPCR as described. (**c**) APAK mRNA levels were measured by qPCR in RKO cells treated either with Luciferase control shRNA (pSMP-Luc 5′- CCCGCCTGAAGTCTCTGATTAA -3′) (Addgene plasmid 36394) (shLuc) or Suv39h1/2 (Suv39h1 (pSMP-Suv39h1 5′-GAGCTCACCTTTGATTACA-3′) (Addgene plasmid 36342), Suv39h2 (pSMP-Suv39h2 5′-CCCGTTACTGCTTCAGCAA-3′) (Addgene plasmid 36344). We thank Dr George Daley, Boston, MA, USA, for depositing these plasmids in Addgene. Cells were exposed to the same hypoxia/reoxygenation treatments as in (**b**). (**d**) APAK mRNA levels were assessed by qPCR in RKO cells treated with Scramble or G9a siRNA (5′-GGACCUUCAUCUGCGAGUA-3′ (Thermo Scientific)), and exposed to the same hypoxia/reoxygenation treatments as in (**b**), *n*=2.

To understand the mechanism of APAK regulation further, we investigated the regulation of H3K9 methyltransferases under different oxygen tensions. H3K9me3 levels were induced in an oxygen-dependent manner in response to hypoxia (Figure 3a).^6^ The oxygen-dependent regulation of H3K9me3 appeared to be HIF-1α independent. The increase in H3K9me3 in hypoxia did not correlate with an obvious increase in expression of the methyltransferases (SETDB1, Suv3h1/2 and G9a), although SETDB1 was moderately increased at the protein level in response to <0.1% O_2_ (Figure 3a). As expected, the mRNA expression of SETDB1, Suv39h2 and G9a was found to be HIF-1/2 independent (Supplementary Figures S3a and b).

The region of hypoxia-induced H3K9me3 enrichment chosen for ChIP-qPCR analysis (Figure 2e) also showed binding of SETDB1 (Supplementary Figure S3c). To test the involvement of this methyltransferase in mediating APAK repression in hypoxia, we treated RKO cells with siRNA to SETDB1. Knockdown of SETDB1 resulted in partial re-expression of APAK in hypoxia, while having no effect on APAK mRNA levels in normoxia or reoxygenation (Figure 3b and Supplementary Figure S3d). SETDB1 can be found in a complex with the transcriptional co-repressor KAP-1, which has a role in heterochromatin formation by promoting H3K9me3 spreading.^26^ The region that we investigated in our ChIP-qPCR analysis also had a KAP-1-binding site (Supplementary Figure S3c). We investigated whether KAP-1 had any effect on APAK repression by again assessing APAK mRNA levels following KAP-1 siRNA-mediated knockdown and found a supportive but nonsignificant effect on APAK expression in hypoxia (Supplementary Figures S3e and f). Next, we investigated whether other methyltransferases, besides SETDB1, could also be mediating APAK repression. Suv39h1 loss did not lead to a significant effect on APAK repression (Figure 3c and Supplementary Figures S3g and h). Finally, we tested the possible involvement of G9a on hypoxia-mediated APAK repression. siRNA-mediated depletion of G9a did not result in a significant effect on APAK repression (Figure 3d and Supplementary Figure S3i). This conclusion was also reached using a specific G9a inhibitor UNC0642, despite the decrease in H3K9me2 and H3K9me3 observed following treatment with increasing concentrations of the inhibitor (Supplementary Figures S3j and k). Therefore, of the principal H3K9 methyltransferases and those tested here, SETDB1 is the primary methyltransferase involved in mediating APAK repression in hypoxia.

### Pharmacological manipulation of H3K9me3 levels affects cell viability and apoptosis in hypoxia

**Figure 4:**
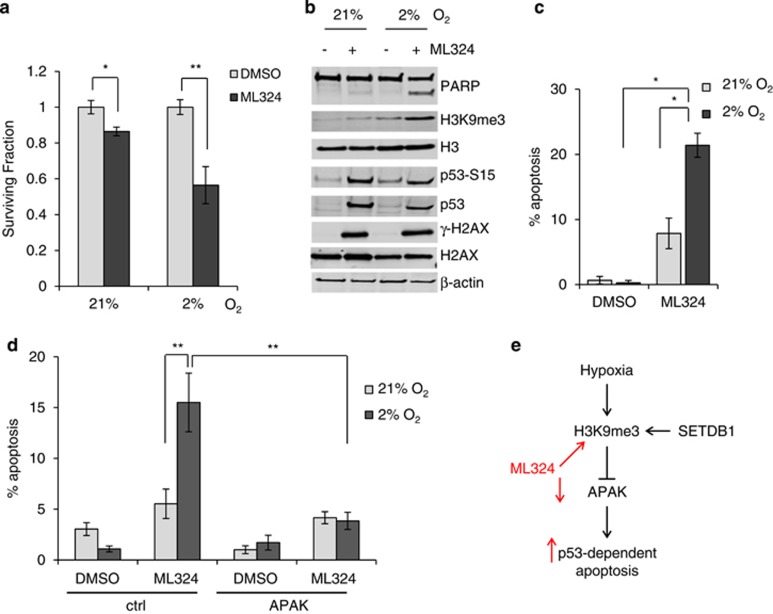
Pharmacological manipulation of H3K9me3 levels affects cell viability and apoptosis in hypoxia. (**a**) RKO cells were treated with 1 μM JMJD2 (JMJD2E) inhibitor, ML324 (Axon Medchem, Groningen, The Netherlands) or DMSO and exposed to 21 or 2% O_2_ for 24 h. Clonogenic survival assays were carried out. Colonies (of at least 50 cells) were allowed to form for 7–10 days. Colonies were then stained with methylene blue and counted. Error bars indicate standard error between technical replicates. A representative graph of one of three independent experiments is shown for all figures showing clonogenic assays unless otherwise stated. (**b**) RKO cells were treated with either DMSO or ML324 (10 μM) and exposed to either 21 or 2% O_2_ for 48 h. Western blotting was carried out with the antibodies indicated as previously described. H2A (Calbiochem, Billerica, MA, USA), γH2AX (Upstate/Millipore) and PARP (Cell Signaling). (**c**) RKO cells were treated as in (**b**). Apoptosis by nuclear morphology was measured as the number of apoptotic/nonapoptotic cells expressed as a percentage of the whole population. (**d**) RKO cells transfected with either Myc-APAK or Myc-empty plasmids and treated with 10 μM ML324 for 24 h followed by incubation in 21% or 2% O_2_ for an additional 24 h. Apoptosis was measured as in (**c**). (**e**) Schematic representation of the proposed model. Hypoxia increases H3K9me3 levels, which in turn leads to APAK repression and p53-dependent apoptosis. Increased H3K9me3 levels and subsequent decreased APAK expression results from both pharmacological inhibition of JMJD2 enzymes (ML324) or through the action of methyltransferase SETDB1.

The data presented so far suggested that APAK is regulated in an H3K9me3- and oxygen-dependent manner and that appropriate regulation of this gene is important for facilitating p53-dependent apoptosis. This led us to question whether pharmacologically manipulating H3K9me3 levels under conditions in which APAK is not normally repressed could also lead to an increase in p53-dependent apoptosis. If this strategy was to be successful, it could be potentially used with therapeutic intent given that low APAK expression appears to correlate with better prognosis in human samples (at least in certain colorectal tumors) (Figure 1d). To test this hypothesis, we used the demethylase inhibitor ML324. First, we asked whether cell viability was affected by ML324 in mildly hypoxic conditions (2% O_2_), which would normally be associated with normal cell growth and proliferation (where APAK levels are not normally repressed). A significant loss of viability was observed in RKO cells exposed to ML324 in hypoxia compared with either hypoxia alone or ML324 treatment in normoxia (Figure 4a and Supplementary Figures S4a and b). We confirmed that H3K9me3 levels had been induced in cells treated with ML324 at 2% O_2_ by western blotting (Figure 4b). Interestingly, treatment with ML324 induced DNA damage, as judged by increased γ-H2AX levels and phosphorylated p53, and this occurred independently of oxygen levels (Figure 4b). These data also demonstrated an increase in PARP cleavage in the cells exposed to ML324 in hypoxic conditions, suggesting that the inhibitor had induced apoptosis (Figure 4b). A significant increase in apoptosis in the presence of ML324 and hypoxia was then further verified (Figure 4c). As the demethylase inhibitor ML324 increases H3K9me3 levels, we predicted that treatment with this inhibitor would result in APAK repression. As expected, APAK mRNA expression was decreased following ML324 treatment (Supplementary Figure S4c). Finally, we asked whether the previously observed ML324-induced apoptosis, which correlated with an increase in H3K9me3 and repression of APAK (Figures 4b and c), was actually dependent on APAK expression. RKO cells either overexpressing APAK or the control vectors were exposed to normoxia or hypoxia (2% O_2_) in the presence or absence of ML324. Once again, ML324 in hypoxia led to increased apoptosis; however, this effect was lost in the presence of overexpressed APAK (Figure 4d and Supplementary Figure S4d). These data suggest that increased apoptosis observed following ML324 treatment was at least partially dependent on APAK expression.

Overall, we provide evidence, for the first time, of a role for H3K9me3 in facilitating p53-dependent apoptosis in response to hypoxia. Specifically, we demonstrate an oxygen-dependent but HIF-1/p53-independent induction of H3K9me3 in response to hypoxia that in turn causes the SETDB1-dependent repression of the p53-negative regulator, APAK and increased apoptosis (Figure 4e). Importantly, this mechanism of gene repression is reversible, meaning that cells that survive periods of severe hypoxia can then restore APAK-mediated control of p53 when normal oxygen levels are restored. Hypoxia-mediated repression of DNA repair pathways can result in the induction of a mutator phenotype and thereby promote tumorigenesis. Indeed, a role for hypoxia in the aberrant epigenetic silencing of genes involved in tumor suppression has been proposed. Under conditions of severe hypoxia, however, the activation of the DNA damage response has been proposed to mount a barrier to tumorigenesis.^6, 27^ Here, we propose that the role of hypoxia-induced H3K9me3 in repressing APAK may actually contribute to such tumorigenesis barrier by facilitating p53-dependent apoptosis. This is supported by the finding that low APAK expression correlated with better prognosis in a small number of colon cancer patients (Figure 1d). It is important to note that the correlation between low APAK expression and a better prognosis would be predicted to be restricted to tumors with wild-type p53.

As a result of the role identified here in hypoxia-induced p53-dependent apoptosis, H3K9me3 can be considered as a potential therapeutic target. We tested the hypothesis that by increasing the levels of H3K9me3, through inhibition of key demethylases, we could drive cells into apoptosis (Figure 4). The inhibitor used in our study, ML324, not only increased the levels of H3K9me3 in cells exposed to mild hypoxia, which normally show a minimal induction of H3K9me3, but it also induced DNA damage. It is likely that not all demethylase inhibitors will have both of these functions and may therefore need to be combined with standard DNA damaging agents to achieve the effects reported here. Furthermore, although ML324 has been reported to be a potent and selective inhibitor of JMJD2, with submicromolar inhibitory activity toward JMJD2E, it would be important to verify whether the use of this inhibitor further contributes to APAK repression via the inhibition of other JMJD2 family members besides JMJD2E.^19^ It is clear from our study, however, that the demethylase inhibitor used here can be exploited to alter gene expression patterns and that these effects are determined by the tumor microenvironment. This further highlights the need to test new agents in settings mimicking conditions such as tumor hypoxia.

In summary, this study highlights a previously uncharacterized role for APAK in response to severe hypoxia, and it provides a novel mechanism for its regulation under these conditions. H3K9me3-mediated regulation of APAK may have important consequences for the maintenance of genomic integrity and the barrier to tumorigenesis.
