# Supplementary material for: H3K9me3 facilitates hypoxia-induced p53-dependent apoptosis through repression of APAK
Source: Oncogene. 2015 May 11;35(6):793–9. doi: 10.1038/onc.2015.134 (PMC4753255; doi:10.1038/onc.2015.134)
Supplement: Supplementary Figures [file onc2015134x1.ppt]

## Slide 1
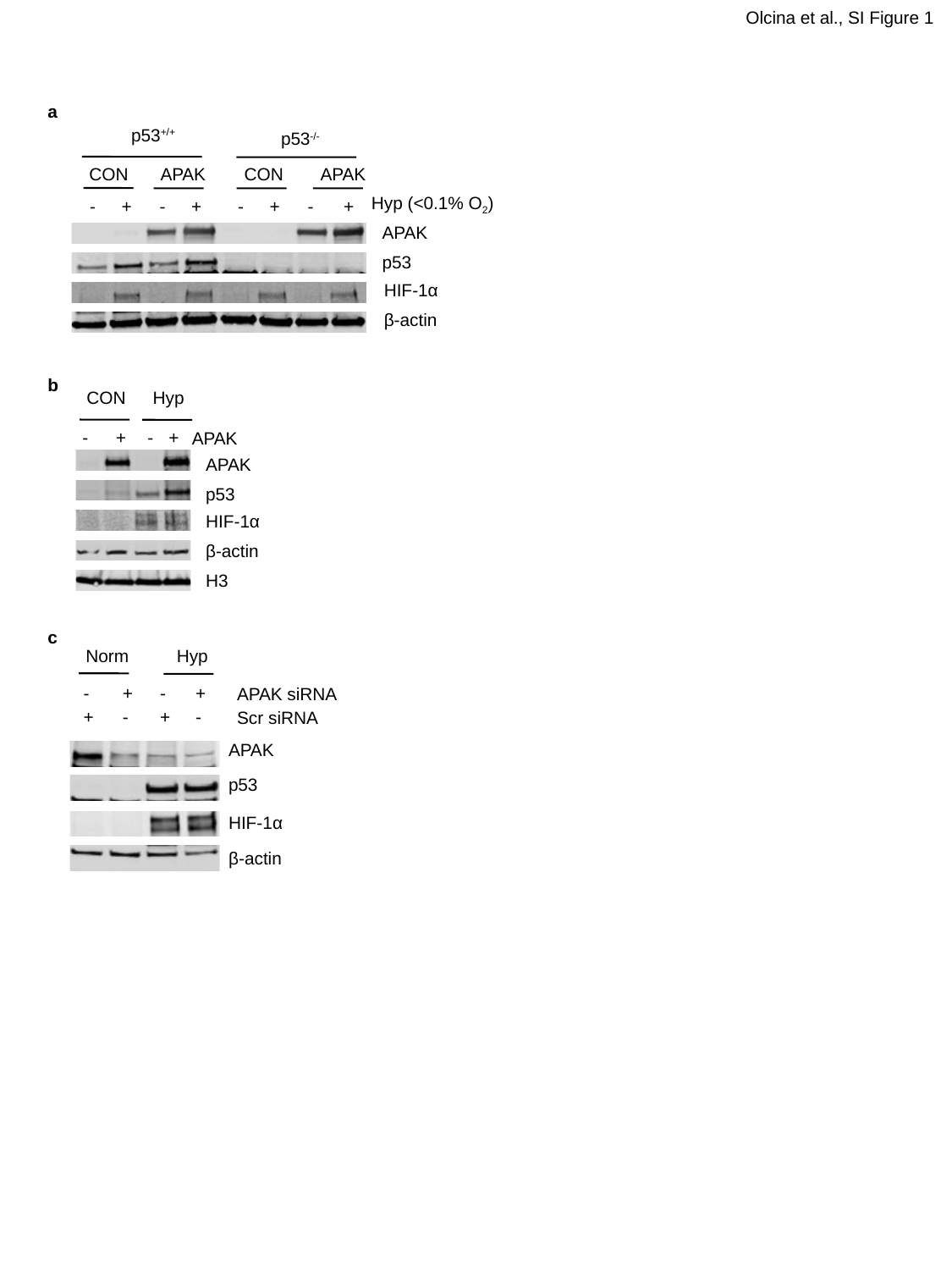

Olcina et al., SI Figure 1
a
p53+/+
p53-/-
CON
APAK
CON
APAK
Hyp (<0.1% O2)
-
+
-
+
-
+
-
+
APAK
p53
HIF-1α
β-actin
b
CON
Hyp
-
+
-
+
APAK
APAK
p53
HIF-1α
β-actin
H3
c
Norm
Hyp
-
+
-
+
APAK siRNA
+
-
+
-
Scr siRNA
APAK
p53
HIF-1α
β-actin

## Slide 2
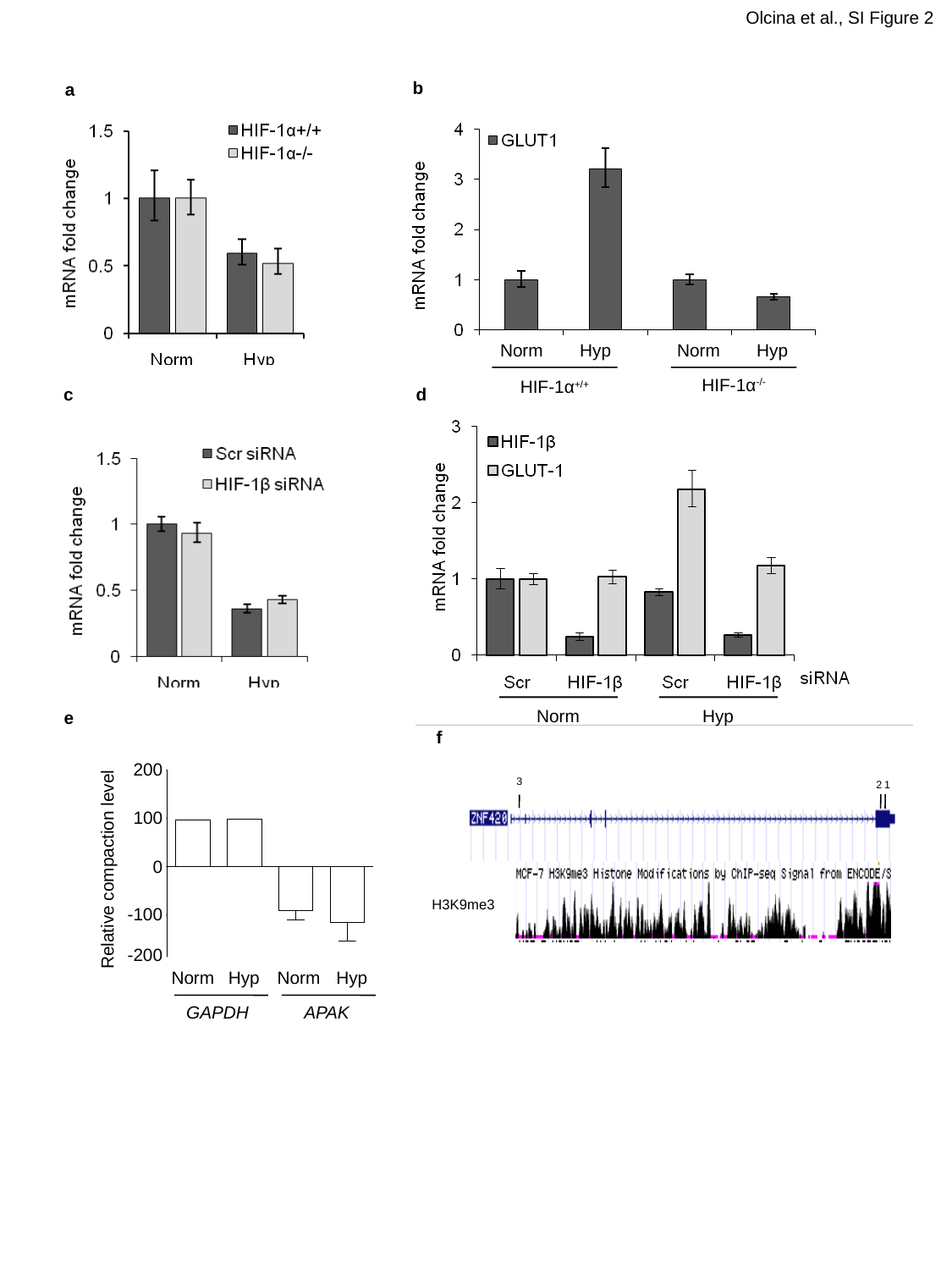

Olcina et al., SI Figure 2
b
a
Norm
Hyp
Norm
Hyp
HIF-1α-/-
HIF-1α+/+
c
d
Norm
Hyp
e
f
200
100
0
Relative compaction level
-100
-200
Norm
Hyp
Norm
Hyp
GAPDH
APAK
3
2
1
H3K9me3

## Slide 3
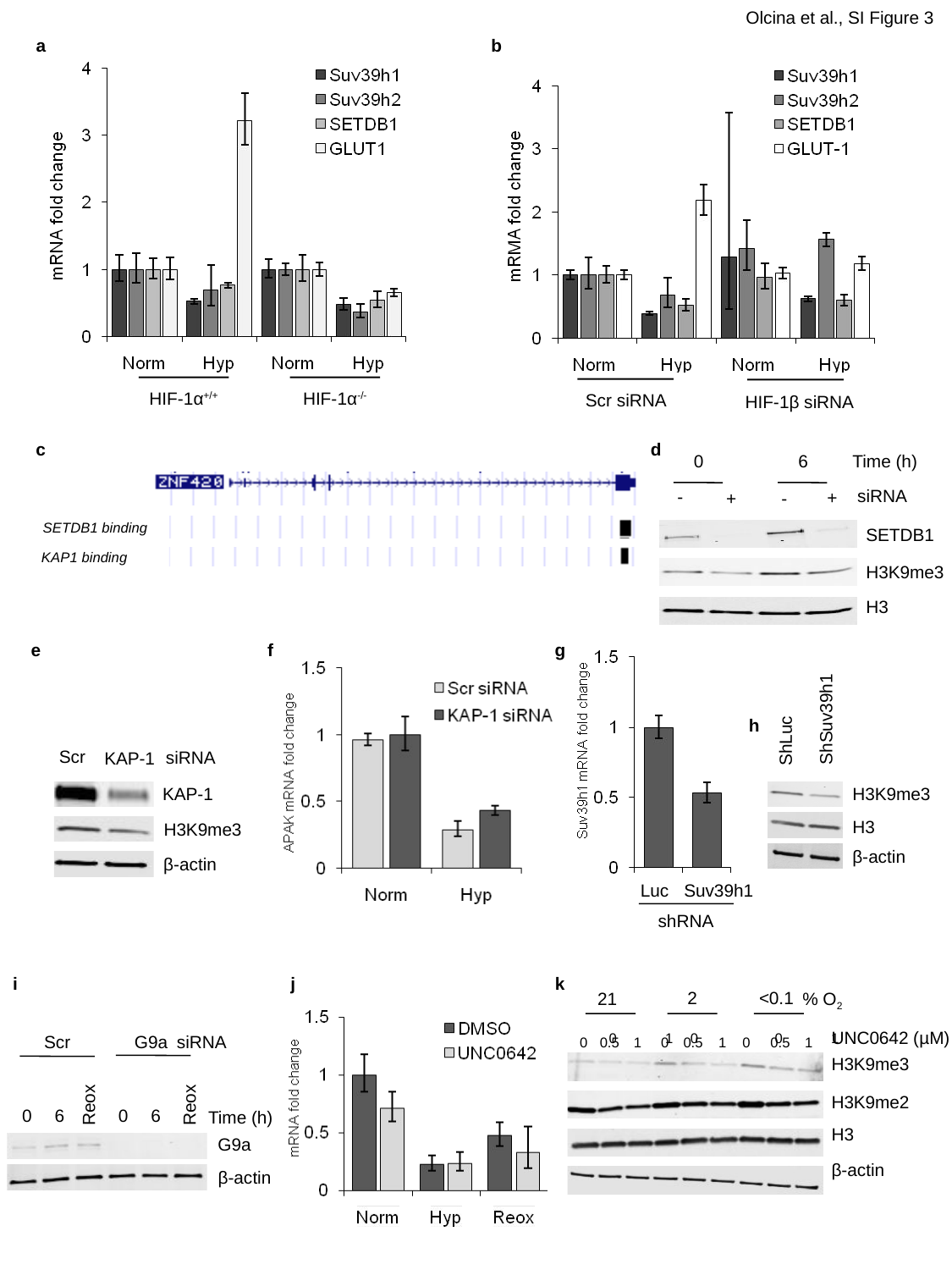

Olcina et al., SI Figure 3
a
b
HIF-1α+/+
HIF-1α-/-
Scr siRNA
HIF-1β siRNA
c
d
 0
 6
Time (h)
 siRNA
-
+
-
+
SETDB1
H3K9me3
H3
SETDB1 binding
KAP1 binding
e
f
g
Luc
Suv39h1
shRNA
ShSuv39h1
ShLuc
H3K9me3
H3
β-actin
h
Scr
siRNA
KAP-1
KAP-1
H3K9me3
β-actin
i
j
k
2
<0.1
% O2
21
UNC0642 (µM)
0
1
0
0
1
1
0
1
0
0.5
0
0.5
1
0.5
H3K9me3
H3K9me2
H3
β-actin
Scr
G9a
siRNA
 Reox
 Reox
0
0
6
6
Time (h)
G9a
β-actin

## Slide 4
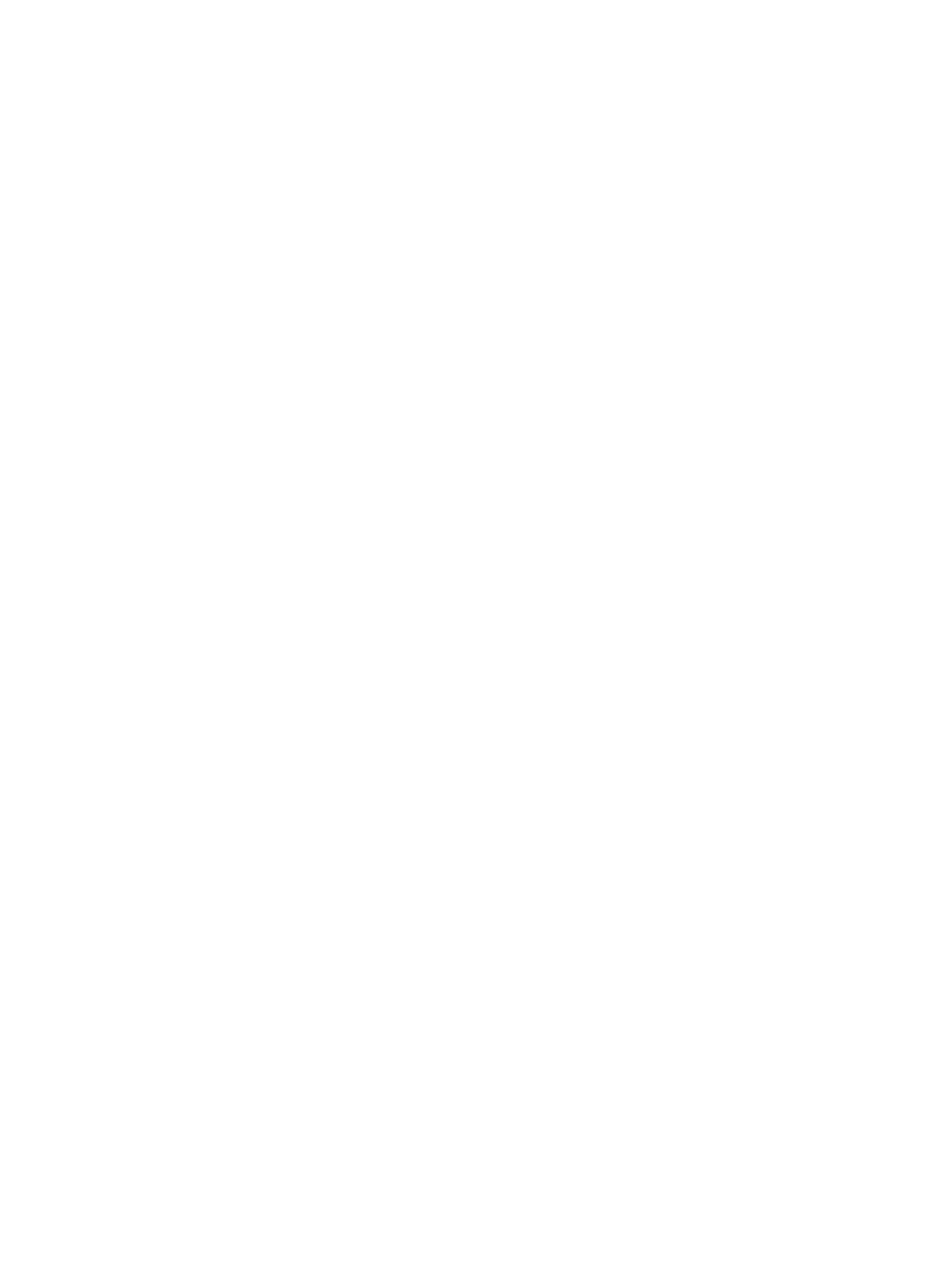

#
