# Supplementary material for: H3K9me3 facilitates hypoxia-induced p53-dependent apoptosis through repression of APAK
Source: Oncogene. 2015 May 11;35(6):793–9. doi: 10.1038/onc.2015.134 (PMC4753255; doi:10.1038/onc.2015.134)
Supplement: Supplementary Tables [file onc2015134x2.docx]

**Supplementary Figures**

**Supplementary Figure S1 Validation of APAK overexpression or siRNA mediated knockdown.**

(a) Myc-tagged-APAK or Myc-empty plasmids were transfected into HCT116 p53^+/+^ or p53^-/-^ cells. These cells were then exposed to 24 h of either Norm (21% O_2_) or Hyp (<0.1% O_2_). Western blotting was carried out as indicated in the main text. APAK (Sigma, Prestige antibodies), p53-S15 (Cell Signaling), HIF-1α (BD-Biosciences) and β-actin (Santa Cruz).

(b) Myc-tagged-APAK or Myc-empty plasmids were transfected into RKO cells. These cells were then exposed to 18 h of either Norm or Hyp. Western blotting was carried out as indicated in the main text. H3 (Cell Signaling).

(c) RKO cells were treated with Scramble (Scr) (Stealth RNAi negative control (Invitrogen)) or APAK siRNA and exposed to 18 h of Norm or Hyp. Western blotting was carried out as previously described with the indicated antibodies.

**Supplementary Figure S2 HIF-1 independence of APAK mRNA expression and assessment of chromatin compaction.**

(a) APAK mRNA levels were measured by qPCR in RKO HIF-1α^+/+^ and HIF1α^-/-^ cells exposed to Norm (21% O_2_) or Hyp (<0.1% O_2_ - 18 h).

(b) The mRNA levels of GLUT1 were measured by qPCR in RKO HIF-1α^+/+^ and HIF-1α^-/-^ cells exposed to 18 h of Norm (21% O_2_) or Hyp (<0.1% O_2_) as a control for both hypoxic treatment and HIF-1α knockdown.

Primers: *GLUT1* forward: ATACTCATGACCATCGCGCTAG, reverse: AAAGAAGGCCACAAAGCCAAAG;

(c) RKO cells were treated with Scramble (Scr) siRNA or HIF-1β siRNA (UGUUUGUCAACCAACAGAGUdtdt, (Thermo Scientific)) and exposed to 18 h of Norm (21% O_2_) or Hyp (<0.1% O_2_). APAK mRNA levels were assessed by qPCR.

(d) RKO cells treated with Scr or HIF-1β siRNA and exposed to the same treatments as in (c). mRNA levels of *HIF-1β* and GLUT1 were measured by qPCR as described above to assess the level of HIF-1β knockdown following HIF-1β siRNA.

Primers: *HIF-1β* forward: GACTACTGCCAACCCCGAAA, reverse: CTCTGGACAATGGCTCCTCC).

(e) RKO cells were exposed to 0 or 6 h of <0.1% O_2_. Quantitative assessment of chromatin structure was performed using an EpiQ chromatin analysis assay (Bio-Rad Laboratories) according to the manufacturer’s protocol. The qPCR primers used as controls for inaccessible (*RHO*) and accessible (*GAPDH*) chromatin regions were provided by the manufacturer and the *APAK* primers used were the same as for ChIP-qPCR analysis shown in Figure 2e. APAK chromatin accessibility was compared to the constitutively accessible GAPDH gene region.

(f) Track of H3K9me3 binding along *APAK* from UCSC genome browser (GRCh37/hg19 assembly). The approximate location of the amplicons for the ChIP primers used in Figure 2 e/f/g are shown along the gene denoted 1, 2 and 3 respectively.

**Figure S3 Regulation of APAK mRNA expression by SETDB1, KAP-1, SUV39h1 and G9a**

(a) The mRNA levels of *Suv39h1*, *Suv39h2*, *SETDB1* and *GLUT1* were measured by qPCR in RKO HIF-1α ^+/+^ and HIF1α ^-/-^cells exposed to Norm (21% O_2_) or Hyp (<0.1% O_2_ - 18 h) as described.

Suv39h1 forward: ATCCGCGAACAGGAATATTACC, reverse: GAGGATACGCACACACTTGAGATT; Suv39h2 forward: CTGATGAGTTCACAGTGGATGCA, reverse: TTTGGGTCACAACTATGATTCACA; SETDB1 forward: ACATCCTCAGCCTCTGCACT, reverse: TTCCAGTACCGGTCAGATCC ;*18S* rRNA forward: TAGAGGGACAAGTGGCGTTC, reverse: CGGACATCTAAGGGCATCAC.

(b) RKO cells were treated with Scramble (Scr) siRNA or HIF-1β siRNA and exposed to 18 h of Norm or Hyp. mRNA levels of Suv39h1, Suv39h2, and SETDB1 were measured by qPCR as described.

(c) Track of SETDB1 and KAP1 binding along *APAK* locus as shown on UCSC genome browser (assembly used: GRCh37/hg19)**.**

(d) Validation of SETDB1 siRNA mediated knockdown from Figure 3b. Western blotting was carried out with the indicated antibodies following treatment of RKO cells with Scr or SETDB1 siRNA and exposure to <0.1% O2 for 0 or 6 h. H3 was used as loading control.

(e) Western blotting verifying siRNA knockdown of KAP-1. Levels of H3K9me3 and total KAP-1 (Bethyl/Universal Biologicals) were used to assess KAP-1 siRNA efficiency. β-actin was used as loading control.

(f) RKO cells were treated with Scramble or KAP-1 siRNA (sequence: GGCCCUAUUCUGUCACGAAtt, Applied Biosystems) and exposed to 6 h of Norm or Hyp (<0.1% O_2_). APAK mRNA levels were assessed by qPCR as described, n = 2.

(g) qPCR was carried out to verify shRNA knockdown of Suv39h1 shown in Figure 3c.

(h) Western blotting was carried out with the indicated antibodies. Levels of H3K9me3 were assessed as a readout of Suv39h1 knockdown from figure 3c. β-actin was used as loading control.

(i) Western blotting with the indicated antibodies to verify siRNA knockdown of G9a shown in Figure 3d. β-actin was used as loading control.

(j) RKO cells were treated for 96 hours with G9a inhibitor (1 μM UNC0642, Structural Genomics Consortium, Toronto), before starting the hypoxic or reoxygenation treatments as in (c). qPCR was used to determine *APAK* mRNA expression as previously described, n =2

(k) RKO cells were treated with UNC0642 (for 96 hours before exposure to hypoxia) at the indicated range of concentrations and exposed to 21%, 2%, or <0.1% O_2_. Western blotting was carried out as indicated. β-actin was used as loading control.

**Figure S4. Pharmacological manipulation H3K9me3 levels impact cell viability and apoptosis in hypoxia**

(a) RKO cells were treated with 1 μM ML324 inhibitor or DMSO and exposed to 2, or (b) 0.1% O_2_ for the indicated time periods. Clonogenic survival assays were carried out as described.

(c) RKO cells were treated with DMSO or 10 μM of ML324 for 48 h under 2% O_2_ conditions and APAK mRNA expression was assessed by qPCR.

(d) RKO cells transfected with either Myc-APAK or Myc-empty plasmids and treated with 10 μM ML324 for 24 h followed by incubation in 21% or 2% O_2_ for additional 24 h. Western blotting was carried out with the indicated antibodies.
